# Measuring residents’ perceptions of social-ecological healthy communities: development and initial psychometric evaluation of a multidimensional scale

**DOI:** 10.3389/fpubh.2026.1957359

**Published:** 2026-10-07

**Authors:** Xinyu Liu, Zhengmei Liao, Tianqi Zhang, Yang Wang, Qiufeng Gu, Jiajun Feng, Hui Xu, Danni Wang

**Affiliations:** 1 Department of Health Promotion and Behavioral Sciences, School of Public Health, Anhui Medical University, Hefei, Anhui, China; 2 Department of General Medicine, Daoxiangcun Street Community Health Service Center, Hefei, Anhui, China; 3 Anhui Cancer Institute, Hefei, Anhui, China

**Keywords:** community health promotion, healthy community, psychometric evaluation, scale development, social-ecological model

## Abstract

**Introduction:**

Effective community health promotion requires information about residents’ health-related characteristics and the interpersonal, environmental, organizational, and policy supports they perceive. The Multidimensional Assessment Scale for Social-Ecological Healthy Communities MASE-HC was designed as a multidimensional resident-reported instrument, not as a unidimensional measure or an objective community-level rating. We report its development and initial psychometric evaluation.

**Methods:**

Items were generated through literature review, a two-round Delphi survey, content-validity assessment, and cognitive interviews. A purposive sample of 412 adult community residents was recruited from one community health service center in Anhui Province, China. Subsample 1 (*n* = 206) was used for item analysis and exploratory factor analysis, and Subsample 2 (*n* = 206) was used for confirmatory factor analysis, convergent and discriminant validity assessment, and hypothesis-based associations with self-rated health. Internal consistency was evaluated in the total sample (*n* = 412).

**Results:**

Data from 412 participants were analyzed. EFA identified a six-factor solution for the final 39-item MASE-HC, accounting for 61.411% of the variance. The final CFA specified six correlated first-order factors and did not evaluate a higher-order general factor. After four post-hoc within-factor residual covariances were added, the refined model showed generally acceptable fit (χ^2^/df = 1.741, RMSEA = 0.060, SRMR = 0.0649, CFI = 0.906, TLI = 0.898, and IFI = 0.907); TLI was marginally below 0.90. The Fornell–Larcker criterion was satisfied for all six factors, whereas convergent validity and internal consistency were comparatively weaker for Diet and Nutrition. Self-rated health was positively associated with five of the six subscales.

**Conclusion:**

The MASE-HC showed preliminary support for a six-factor multidimensional structure in the present sample. The Diet and Nutrition subscale requires further refinement, and independent psychometric evaluation and replication in larger, more representative samples are needed before routine use or any community-level comparison.

## 1 Introduction

Community health promotion requires an understanding of not only individual health-related behaviors but also the broader social and environmental conditions that enable or constrain those behaviors (1, 2). The social-ecological model (SEM) provides a useful framework for understanding these multilevel influences on health (3–5). Originally applied to health promotion by McLeroy et al., the SEM proposes that health behaviors and outcomes are shaped by interacting influences at five levels: intrapersonal, interpersonal, organizational, community, and public policy (6). Community health assessment (CHA) is a core public health function that encompasses both a systematic assessment process and its tangible products, such as community health profiles and reports describing population health status, relevant determinants, and available health-related resources (7). It involves the systematic collection and analysis of data to identify priority health needs and issues within a defined population and to inform priority setting, intervention planning, and health-promotion strategies (8). Recent reviews have also highlighted the diversity of community health assessment approaches and tools used across community and practice settings (9, 10). From a social-ecological perspective, such assessment should therefore consider not only individual health-related characteristics but also the interpersonal, environmental, organizational, and policy contexts in which health behaviors occur (11).

The present study conceptualized social-ecological healthy-community assessment as a multidimensional resident-reported framework, rather than as a unidimensional measure of an objective community-level trait (12). The initial content framework comprised four broad domains: Individual Health and Behavior, Interpersonal Support, Built Environment Support, and Organizational Policy Support. These domains were informed by, rather than intended to reproduce one-to-one, the five canonical levels of the SEM (6). Individual Health and Behavior primarily reflects intrapersonal characteristics; Interpersonal Support corresponds to interpersonal influences; Built Environment Support captures residents’ perceptions of salient features of the community physical environment; and Organizational Policy Support reflects organizational support together with residents’ experiences of locally implemented health-promoting policies and services. This grouping was intended to provide a pragmatic resident-level representation of theoretically related social-ecological components while acknowledging that broader community processes, such as collective efficacy and civic participation, are not comprehensively represented.

Several existing instruments assess important but more circumscribed aspects of community health. Neighborhood-based scales have been developed to assess residents’ perceptions of neighborhood characteristics and environmental conditions (13). The Community Efficacy for Non-Communicable Disease Management (COEN) scale assesses a community’s ability to provide non-communicable disease management for its residents (14). The multidimensional Health Resilience Scale assesses health resilience among community-dwelling adults (15), whereas the Healthy Lives 5-Axis Scale (HL-5) is a brief provider-rated instrument developed for individuals with complex health and social needs (16). The Primary Care Needs Assessment (PCNA) questionnaire also combined community input with psychometric validation to assess perceived, unmet, and contextual healthcare needs in primary-care settings (17).

Although these instruments provide valuable measures for their intended constructs and populations, they primarily address specific domains of neighborhood environment, community efficacy, resilience, or multidimensional health rather than integrating individual health-related characteristics with perceived interpersonal, built-environmental, and organizational-policy supports within a single social-ecological assessment framework (13–17). More broadly, a 2024 systematic review identified 47 healthy-lifestyle instruments, found substantial heterogeneity in their domains and psychometric coverage, and concluded that most available assessments required improvement (18). A 2026 systematic review with concept analysis likewise defined healthy living as a multidimensional life-course process integrating behavioral-physical, cognitive-psychological, social-relational, and contextual-structural dimensions (19). The MASE-HC was therefore developed as a complementary multidimensional resident-reported instrument rather than as a replacement for these construct-specific measures (Figure 1).

**Figure 1:**
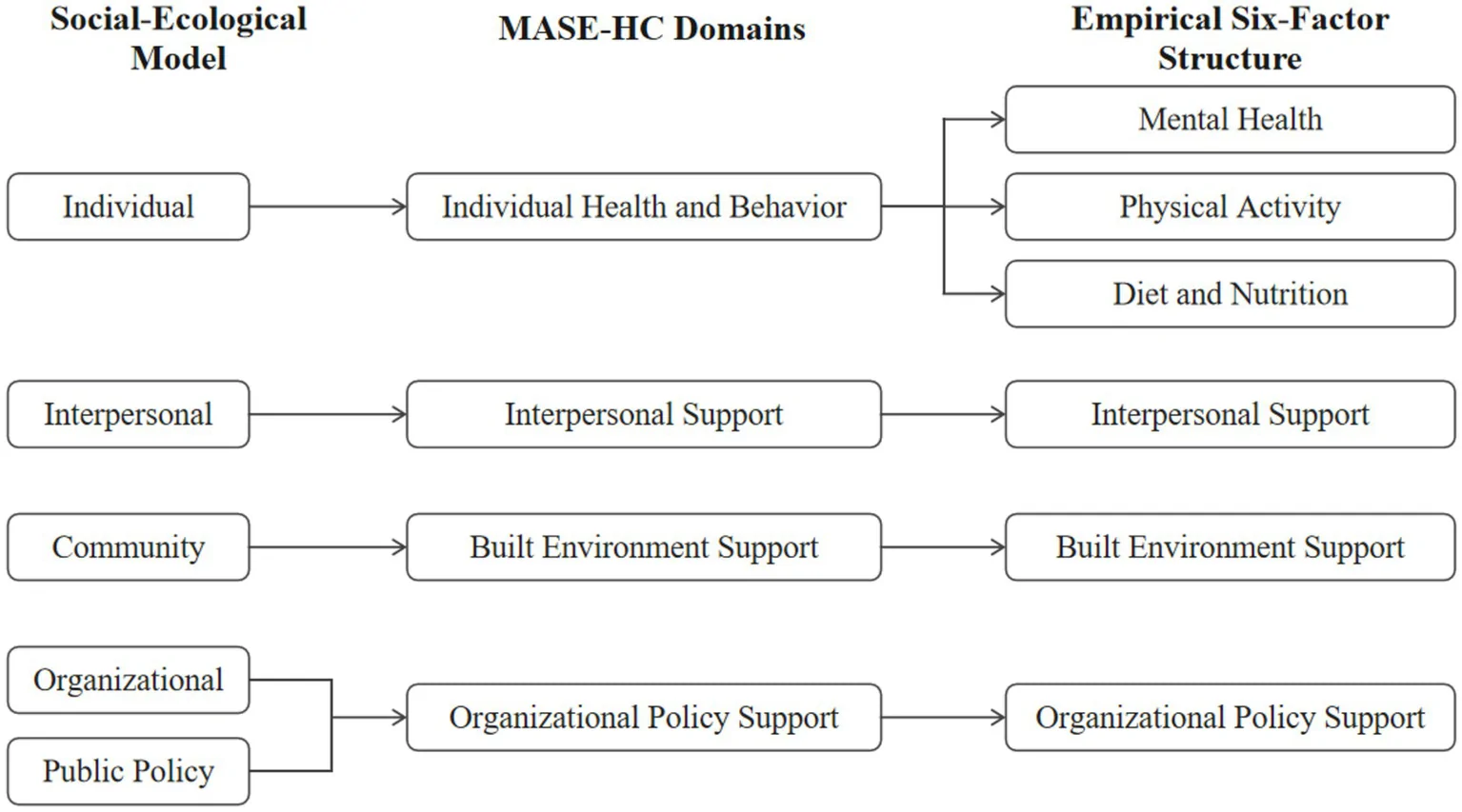
Conceptual mapping of the Social-Ecological Model, MASE-HC domains, and empirical six-factor structure.

Accordingly, this study aimed to develop and conduct an initial psychometric evaluation of the Multidimensional Assessment Scale for Social-Ecological Healthy Communities (MASE-HC). Specifically, we aimed to: (1) generate and refine an initial item pool based on the SEM, relevant literature, expert consensus, and cognitive interviews; (2) examine item performance and the exploratory factor structure of the instrument; (3) evaluate the resulting measurement structure and its internal consistency, convergent validity, and discriminant validity in a confirmatory subsample; and (4) examine hypothesis-based associations with self-rated health as hypothesis-based external construct evidence.

## 2 Methods

This scale development and initial psychometric evaluation study followed the general procedures recommended by DeVellis et al. (20), and was conducted in three phases: item development, exploratory scale development, and initial psychometric evaluation (21–23).

### 2.1 Phase 1 item development

#### 2.1.1 Item generation

An initial item pool was generated through a literature review of community health assessment, healthy community evaluation, and social–ecological health promotion studies published up to August 2025. Items were drafted to reflect four theoretically defined domains derived from the social–ecological model: Individual Health and Behavior, Interpersonal Support, Built Environment Support, and Organizational Policy Support. After reviewing existing instruments and extracting relevant indicators, an initial pool of 122 items was developed.

The MASE-HC uses a 5-point Likert response format. Higher scores indicate more favorable perceived health status or stronger perceived social–ecological support for community health. Positively worded items were scored directly, whereas negatively worded items were reverse-coded using the formula 6 minus the raw score. This scoring procedure ensured that higher scores consistently reflected more favorable health-related characteristics or stronger perceived support. For the final instrument, subscale scores were calculated separately for the six retained factors. A single overall score was not used as the primary interpretive score because the final measurement model specified six correlated first-order factors and did not evaluate a higher-order general factor. The final 39-item instrument is provided in Supplementary File 4.

#### 2.1.2 Item reduction

Item reduction was conducted through a two-round Delphi survey, content validity assessment, and cognitive interviews. The Delphi survey was used to evaluate the relevance, clarity, and theoretical representativeness of the initial items (24). Twenty-one experts in health education and promotion, health policy and management, and public health participated. Items were rated on a 5-point scale from 1 (least important) to 5 (most important). The Delphi process was stopped after consensus was reached. Second, a content validity survey was conducted with a panel of ten experts in public health, who were asked to rate the relevance of each item using a 4-point Likert scale: 1 (not relevant), 2 (somewhat relevant), 3 (relevant), and 4 (very relevant). The item-level content validity index (I-CVI; the proportion of experts rating 3 or 4) and the scale-level content validity index (S-CVI/Ave; the average of the I-CVI scores) were calculated. Items with an I-CVI of at least 0.78 were retained, and the rest were deleted (25, 26). Finally, cognitive interviews were conducted with 20 residents recruited by purposive sampling from a community health service center in Hefei. The interviews assessed item comprehensibility, interpretation consistency, response burden, and completion time. Items were revised when residents reported ambiguity or difficulty in understanding (27).

### 2.2 Phase 2 scale development

#### 2.2.1 Participants

A formal survey was conducted using purposive sampling among adult community residents. Eligible participants were residents aged 18 years or older who had lived in the community for more than 6 months, were able to understand and complete the questionnaire, and provided informed consent. Participants who were unable to communicate effectively or complete the questionnaire were excluded.

Separate samples are preferable for exploratory and confirmatory factor analyses when feasible because they reduce direct reuse of the same observations during exploration and confirmation (22, 28). Required sample size for factor analysis is not determined by a universal participant-to-item ratio; it also depends on features such as communalities, factor overdetermination, and model complexity (29, 30). In this study, the prespecified target of approximately 335 participants was pragmatically based on the 61 items retained after content-validity assessment plus an allowance of approximately 10% for invalid responses. A total of 412 valid responses were obtained and randomly divided into two equal subsamples (*n* = 206 each) for EFA and CFA, respectively.

#### 2.2.2 Data collection

Data were collected from November to December 2025 at a community health service center in a subdistrict of Anhui Province. The study was approved by the Medical Ethics Committee. Participants were recruited through community notices and completed a face-to-face questionnaire after being informed of the study purpose and procedures.

#### 2.2.3 Statistical analysis

Data were analyzed using SPSS 23.0 (SPSS Inc., Chicago, IL, USA), AMOS 26.0 (IBM Corp., Armonk, NY, USA), and R version 4.6.1 (R Core Team, 2026). Continuous variables were summarized as means and standard deviations, and categorical variables were summarized as frequencies and percentages. The total sample was randomly divided into two equal subsamples. Subsample 1 (*n* = 206) was used for item analysis and exploratory factor analysis (EFA), whereas Subsample 2 (*n* = 206) was used for confirmatory factor analysis (CFA) to test the proposed measurement model, convergent and discriminant validity assessment, and associations with self-rated health. No missing responses were present among the 50 items entered into the first EFA or among the final 39 retained items in the total sample; consequently, no imputation was performed and no participant was excluded from EFA, CFA, or reliability analysis because of missing item data.

#### 2.2.4 Item analysis

Item analysis was conducted in Subsample 1 (*n* = 206). Item performance was evaluated using the critical-ratio method, corrected item–domain correlations, and changes in internal consistency after item deletion. For the critical-ratio method, participants were divided into high- and low-scoring groups using the upper and lower 27% of the corresponding domain scores, and item scores were compared between the two groups (31, 32). Items were considered for retention when the critical-ratio statistic was ≥ 3.0, the between-group difference was statistically significant (*p* < 0.05), and the corrected item–domain correlation was > 0.30 (33). Changes in Cronbach’s α after item deletion were also examined. Item-removal decisions were based on the overall pattern of statistical evidence together with conceptual relevance and content coverage, rather than on any single criterion alone (21, 22).

#### 2.2.5 Exploratory factor analysis

Exploratory factor analysis (EFA) was conducted in Subsample 1 (*n* = 206) using principal axis factoring (PAF) with Direct Oblimin rotation because correlations among the underlying factors were expected (34–36). Sampling adequacy was assessed using the Kaiser–Meyer–Olkin (KMO) statistic and Bartlett’s test of sphericity, with a KMO value > 0.80 considered indicative of adequate sampling adequacy (37, 38). Factor retention was primarily informed by parallel analysis based on Pearson correlations, using 1,000 random datasets and the 95th percentile of simulated common-factor eigenvalues as the comparison criterion (39, 40). Scree-plot inspection, factor-loading patterns, and theoretical interpretability were also considered.

Items with primary factor loadings < 0.40 were considered weak. Items with loadings ≥ 0.30 on more than one factor were examined for cross-loading, and items with a difference < 0.20 between their two largest absolute loadings were considered to show a complex loading pattern. Factors represented by only two items were considered underdefined and were evaluated for substantive and structural adequacy. EFA was repeated iteratively after removal of poorly performing items until a stable and interpretable factor solution was obtained. A cumulative variance explained of > 50% was considered supportive of an interpretable factor solution (41).

### 2.3 Phase 3 initial psychometric evaluation

#### 2.3.1 Confirmatory factor analysis

Confirmatory factor analysis was conducted in Subsample 2 (*n* = 206) using AMOS 26.0 and maximum-likelihood estimation. The five-category items were treated as approximately continuous in this maximum likelihood (ML) analysis. Methodological evidence indicates that the performance of continuous-data estimators with ordinal indicators depends on the number and distribution of response categories (42, 43). The final CFA specified six correlated first-order factors and did not evaluate a higher-order general factor. Model fit was assessed using the chi-square-to-degrees-of-freedom ratio (χ^2^/df), the Root Mean Square Error of Approximation (RMSEA), the Standardized Root Mean Square Residual (SRMR), the Comparative Fit Index (CFI), the Tucker–Lewis Index (TLI), and the Incremental Fit Index (IFI). Values of χ^2^/df < 3.0, RMSEA < 0.08, SRMR < 0.08, and incremental fit indices around or above 0.90 were treated as approximate guides and interpreted collectively rather than as absolute pass/fail rules (44, 45).

The initial correlated six-factor model was evaluated first. Modification indices were then inspected for localized misfit. Four residual covariances were added *post hoc*, all between items within the same latent factor, when a comparatively large modification index was accompanied by plausible overlap in item content (46, 47). The refined model was re-estimated and compared with the initial specification; no cross-factor residual covariance or higher-order factor was introduced.

#### 2.3.2 Convergent and discriminant validity

Convergent validity was evaluated using standardized factor loadings, composite reliability (CR), and average variance extracted (AVE) derived from the CFA model. CR values ≥ 0.70 and AVE values ≥ 0.50 were considered indicative of adequate convergent validity (48, 49). Values close to these thresholds were interpreted cautiously in conjunction with factor loadings and the conceptual coherence of the factor.

Discriminant validity was assessed using the Fornell–Larcker criterion. Discriminant validity was considered supported when the square root of the AVE for each factor exceeded its correlations with the other latent factors (49). Satisfaction of this criterion was interpreted as criterion-specific evidence rather than definitive proof of discriminant validity because the Fornell-Larcker procedure can have limited sensitivity for detecting insufficient construct separation (50).

#### 2.3.3 Hypothesis-based external construct validity

Because no established external criterion is available for the multidimensional construct assessed by the MASE-HC, self-rated health was examined as a hypothesis-based external construct rather than as a criterion measure. Participants rated their overall health using a single 5-point item (51, 52). Spearman rank correlations were calculated between self-rated health and the six MASE-HC subscale scores in the confirmatory subsample (*n* = 206), because the self-rated health item was ordinal. The direction, magnitude, and statistical significance of the associations were examined as supplementary evidence of external construct validity. These associations were interpreted cautiously because self-rated health primarily reflects individual health status and does not directly represent all interpersonal, environmental, organizational, or policy components assessed by the MASE-HC.

#### 2.3.4 Tests of reliability

Internal consistency was assessed using Cronbach’s α, McDonald’s ω, and split-half reliability with the Spearman–Brown coefficient. Cronbach’s α values >0.70 were considered acceptable, and values >0.80 were considered excellent (20). McDonald’s ω was also computed, with values > 0.70 considered to reflect adequate internal consistency reliability (53). Reliability coefficients were calculated for each of the six retained subscales and, descriptively, for the total 39-item set.

## 3 Results

### 3.1 Sample characteristics

A total of 430 questionnaires were distributed, of which 412 were included in the analysis after 18 incomplete or otherwise invalid questionnaires were excluded, yielding a valid-questionnaire proportion of 95.81%. The valid sample was randomly divided into two equal subsamples using a random number generator. Subsample 1 (*n* = 206) was used for item analysis and EFA, whereas Subsample 2 (*n* = 206) was used for CFA and construct-validity analyses. The total sample (*n* = 412) was used for reliability analysis. Participant characteristics are summarized in Table 1.

**Table 1:** Participant characteristics of the total sample and two subsamples.

Characteristics	Total sample (*n* = 412)	Subsample 1 (*n* = 206)	Subsample 2 (*n* = 206)
Gender, *n* (%)			
Male	262 (63.6)	126 (61.2)	136 (66.0)
Female	150 (36.4)	80 (38.8)	70 (34.0)
Age, years, mean ± SD	33.87 ± 10.26	33.57 ± 9.93	34.17 ± 10.60
Education level, *n* (%)			
Primary school or below	4 (1.0)	0 (0)	4 (1.9)
Middle school	20 (4.9)	10 (4.9)	10 (4.9)
High school	39 (9.5)	18 (8.7)	21 (10.2)
Associate degree	113 (27.4)	52 (25.2)	61 (29.6)
Bachelor’s degree	208 (50.5)	113 (54.9)	95 (46.1)
Master’s degree or above	28 (6.8)	13 (6.3)	15 (7.3)
Living arrangement, *n* (%)			
Living alone	65 (15.8)	34 (16.5)	31 (15.0)
Not living alone	347 (84.2)	172 (83.5)	175 (85.0)

### 3.2 Item development

The initial item pool contained 122 items. After two rounds of the Delphi survey, 58 items were deleted, and three additional items were consolidated with overlapping items, leaving 61 items. The I-CVI values of the retained items ranged from 0.80 to 1.00, and the S-CVI/Ave was 0.968, indicating acceptable content validity. During the cognitive interviews, participants reported no major difficulties in understanding the items, and no further revisions were made. The preliminary field-test version of the MASE-HC therefore consisted of 61 items across four domains: Individual Health and Behavior (29 items), Interpersonal Support (12 items), Built Environment Support (11 items), and Organizational Policy Support (9 items).

### 3.3 Item analysis and exploratory factor analysis

Item analysis was conducted sequentially in Subsample 1 (*n* = 206). Critical-ratio analysis of all 61 field-test items led to removal of 10 items (A1, A2, A14, A19, A20, A23, A24, A26, A28, and A29), leaving 51 items for corrected item–domain correlation analysis. A3 was then removed because its corrected item–domain correlation was 0.207. Cronbach’s α if item deleted was examined for the remaining 50-item set and did not lead to further removal. The resulting 50 items were retained for exploratory factor analysis. Detailed results for the 61-, 51-, and 50-item stages are provided in Supplementary File 1.

Exploratory factor analysis was conducted iteratively using principal axis factoring (PAF) with Direct Oblimin rotation. Parallel analysis supported a six-factor solution, with the observed common-factor eigenvalues exceeding the 95th-percentile simulated eigenvalues through the sixth factor but not the seventh (Supplementary File 2). This six-factor solution was therefore examined throughout the subsequent item-refinement process.

In the first EFA, 50 items were entered into the analysis. The Kaiser–Meyer–Olkin (KMO) measure was 0.907, and Bartlett’s test of sphericity was significant, χ^2^(1225) = 7705.492, *p* < 0.001, supporting the factorability of the correlation matrix. The six-factor solution accounted for 54.433% of the variance. A21 and A22 showed weak factor loadings, B11 and B12 formed an underdefined two-item factor, and C10 showed substantial cross-loading; these five items were removed.

The second EFA was conducted on the remaining 45 items. Sampling adequacy remained high (KMO = 0.912), and Bartlett’s test remained significant, χ^2^(990) = 7072.545, *p* < 0.001. The six-factor solution explained 57.646% of the variance. A15 showed a weak and complex loading pattern, whereas A25 and C11 had primary loadings below 0.40 and were removed. A27 was subsequently removed because it consistently loaded with Mental Health and did not support a distinct additional factor.

The third EFA was therefore conducted on 41 items. The KMO value was 0.915, and Bartlett’s test of sphericity was significant, χ^2^(820) = 6694.592, *p* < 0.001. The six-factor solution explained 60.548% of the variance. C9 had a primary loading below 0.40, whereas B5 showed a persistent complex loading pattern, with a secondary loading above 0.30 and a difference of less than 0.20 between its two largest absolute loadings. Both items were removed.

The final EFA was conducted on 39 items. The KMO value increased to 0.916, and Bartlett’s test remained significant, χ^2^(741) = 6375.980, *p* < 0.001. The final six-factor solution accounted for 61.411% of the variance, and all retained items had primary factor loadings ranging from 0.420 to 0.927. The resulting 39-item configuration comprised six factors: Mental Health (6 items), Physical Activity (4 items), Diet and Nutrition (3 items), Interpersonal Support (9 items), Built Environment Support (8 items), and Organizational Policy Support (9 items). The four broader social-ecological domains were retained as conceptual groupings rather than modeled as higher-order latent factors. The iterative item-refinement results are summarized in Table 2. The final six-factor pattern matrix is provided in Supplementary File 3.

**Table 2:** Iterative exploratory factor analysis and item refinement of the MASE-HC.

Analysis stage	No. of items	KMO	Bartlett’s χ^2^ (df)	Variance explained (%)	Items removed / refinement
Initial EFA	50	0.907	7705.492 (1225), *p* < 0.001	54.433	A21, A22, B11, B12, C10
Second EFA	45	0.912	7072.545 (990), *p* < 0.001	57.646	A15, A25, C11; A27 subsequently removed
Third EFA	41	0.915	6694.592 (820), *p* < 0.001	60.548	C9, B5
Final EFA	39	0.916	6375.980 (741), *p* < 0.001	61.411	None

All EFAs were conducted using principal axis factoring with Direct Oblimin rotation. A six-factor solution was retained based on parallel analysis, scree-plot inspection, loading patterns, and theoretical interpretability.

### 3.4 Confirmatory factor analysis, convergent validity, and discriminant validity

The final CFA specified six correlated first-order factors and did not evaluate a higher-order general factor. The initial 39-item correlated six-factor model showed suboptimal fit (χ^2^/df = 2.074, RMSEA = 0.072, SRMR = 0.0656, CFI = 0.863, TLI = 0.852, and IFI = 0.864). Inspection of modification indices identified localized areas of misfit. Four within-factor residual covariances were then added *post hoc* when the corresponding item pairs showed plausible content overlap. The refined model showed improved and generally acceptable fit (χ^2^/df = 1.741, RMSEA = 0.060, SRMR = 0.0649, CFI = 0.906, TLI = 0.898, and IFI = 0.907). TLI was marginally below the conventional 0.90 reference value; therefore, the refined model was interpreted as showing generally acceptable fit rather than as uniformly meeting all conventional fit criteria (Table 3).

**Table 3:** Model-fit indices for the initial and refined six-factor CFA models.

Model	χ^2^/df	RMSEA	SRMR	CFI	TLI	IFI
Initial model	2.074	0.072	0.0656	0.863	0.852	0.864
Refined model	1.741	0.060	0.0649	0.906	0.898	0.907

χ^2^/df, chi-square-to-degrees-of-freedom ratio; RMSEA, Root Mean Square Error of Approximation; SRMR, Standardized Root Mean Square Residual; CFI, Comparative Fit Index; TLI, Tucker-Lewis Index; IFI, Incremental Fit Index.

Standardized factor loadings were examined as part of the assessment of convergent validity (Figure 2). In the refined six-factor model, all retained items had standardized factor loadings greater than 0.40, ranging from 0.407 to 0.945. Composite reliability (CR) values ranged from 0.674 to 0.950, and average variance extracted (AVE) values ranged from 0.417 to 0.681. Mental Health, Interpersonal Support, Built Environment Support, and Organizational Policy Support met the conventional criteria of CR ≥ 0.70 and AVE ≥ 0.50. Physical Activity showed adequate composite reliability (CR = 0.791), while its AVE (0.496) was marginally below the conventional 0.50 threshold. Diet and Nutrition showed comparatively weaker convergent-validity evidence (CR = 0.674; AVE = 0.417), indicating that this factor may require further refinement in future validation studies.

**Figure 2:**
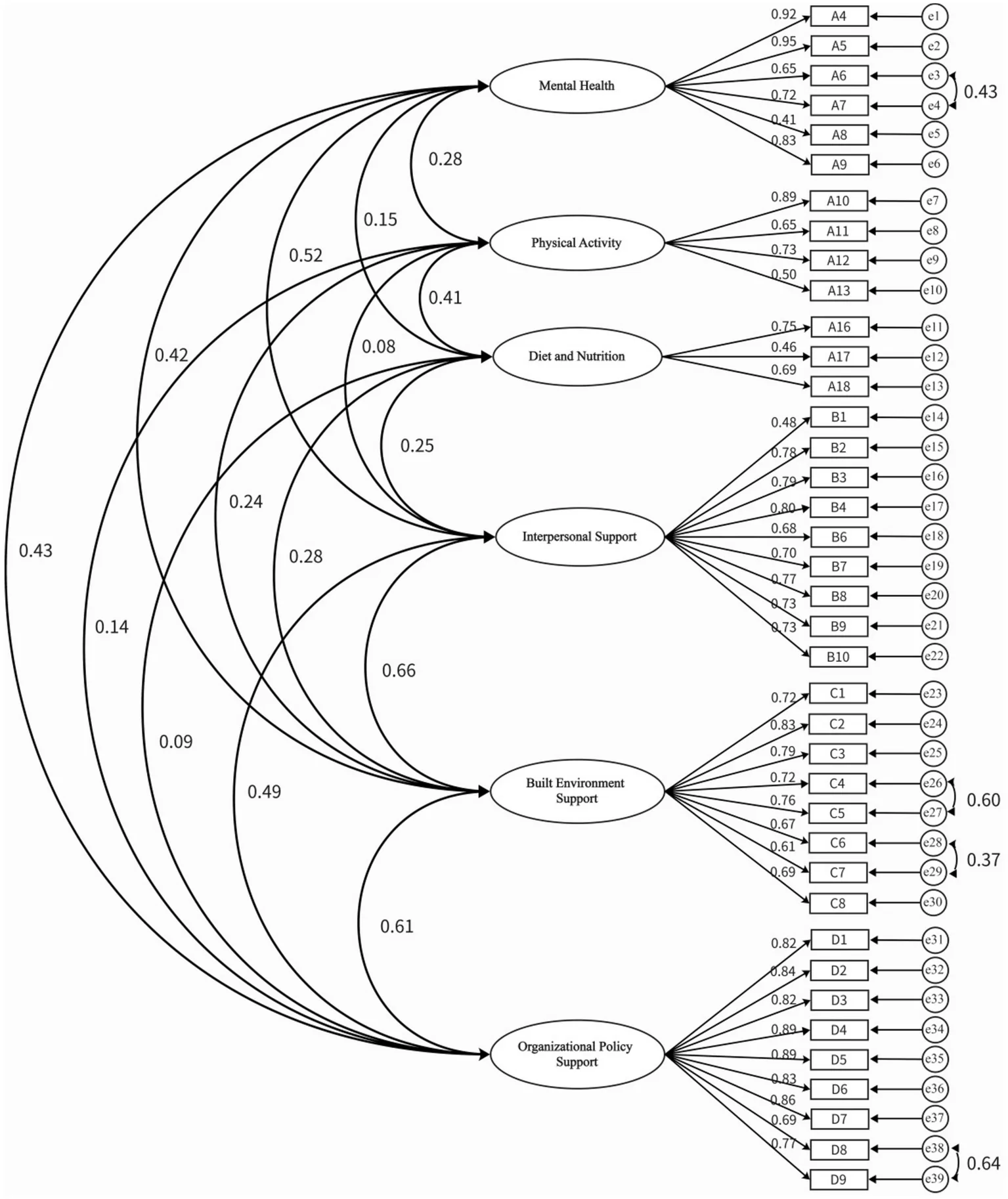
Standardized six-factor CFA model of the 39-item MASE-HC.

Discriminant validity was assessed using the Fornell–Larcker criterion. The square root of the AVE for each factor exceeded its correlations with all other factors, supporting discriminant validity across the six-factor structure. The highest inter-factor correlation was observed between Interpersonal Support and Built Environment Support (*r* = 0.658), which remained below the square root of the AVE for both factors (0.722 and 0.727, respectively). Overall, discriminant validity was supported across all six factors. Convergent-validity evidence was generally satisfactory for most factors; however, the AVE for Physical Activity was marginally below the conventional 0.50 threshold (AVE = 0.496), while Diet and Nutrition showed comparatively weaker convergent validity (CR = 0.674; AVE = 0.417), indicating that this subscale may require further refinement in future validation studies (Tables 4, 5).

**Table 4:** Convergent-validity estimates for the refined six-factor MASE-HC.

Subscale	Items	CR	AVE	$\sqrt{\mathrm{AVE}}$
Mental Health	6	0.888	0.584	0.764
Physical Activity	4	0.791	0.496	0.704
Diet and Nutrition	3	0.674	0.417	0.646
Interpersonal Support	9	0.906	0.522	0.722
Built Environment Support	8	0.899	0.528	0.727
Organizational Policy Support	9	0.950	0.681	0.825

CR, composite reliability; AVE, average variance extracted; $\sqrt{\mathrm{AVE}}$ , square root of the average variance extracted.

**Table 5:** Discriminant validity of the refined six-factor MASE-HC.

Subscale	Mental health	Physical activity	Diet and nutrition	Interpersonal support	Built environment support	Organizational policy support
Mental Health	**0.764**					
Physical Activity	0.276	**0.704**				
Diet and Nutrition	0.151	0.405	**0.646**			
Interpersonal Support	0.521	0.075	0.249	**0.722**		
Built Environment Support	0.418	0.235	0.277	0.658	**0.727**	
Organizational Policy Support	0.432	0.143	0.087	0.486	0.611	**0.825**

Diagonal values in bold are $\sqrt{\mathrm{AVE}}$ ; off-diagonal values are inter-factor correlations.

### 3.5 Associations with self-rated health

Self-rated health was examined as a supplementary external construct rather than as a criterion measure. Participants rated their overall health on a 5-point scale. Associations between self-rated health and the six MASE-HC factor scores were examined to assess whether the observed pattern was broadly consistent with theoretical expectations. Given that self-rated health primarily reflects individual health status rather than contextual community support, these associations were interpreted as supplementary construct evidence only.

Spearman rank correlations were calculated between self-rated health and the six MASE-HC subscale scores in the confirmatory subsample (*n* = 206), with *rₛ* denoting Spearman’s rank correlation coefficient. Self-rated health showed statistically significant positive associations with five of the six subscales, with the strongest association observed for Mental Health (*rₛ* = 0.340, *p* < 0.001), followed by Interpersonal Support (*rₛ* = 0.267, *p* < 0.001), Organizational Policy Support (*rₛ* = 0.183, *p* = 0.008), Built Environment Support (*rₛ* = 0.175, *p* = 0.012), and Physical Activity (*rₛ* = 0.172, *p* = 0.014). The association with Diet and Nutrition was not statistically significant (*rₛ* = 0.039, *p* = 0.578). Across the six comparisons, the coefficients ranged from 0.039 to 0.340. Overall, the observed associations were weak to moderate in magnitude according to commonly used interpretive conventions for correlation coefficients (54), and provided only limited supplementary evidence of external construct validity. Because self-rated health was assessed using a single item and does not fully represent the multidimensional construct assessed by the MASE-HC, these findings should be interpreted cautiously (Table 6).

**Table 6:** Spearman correlations between MASE-HC subscales and self-rated health.

Subscale	Spearman’s rₛ	*p*
Mental Health	0.340	<0.001
Physical Activity	0.172	0.014
Diet and Nutrition	0.039	0.578
Interpersonal Support	0.267	<0.001
Built Environment Support	0.175	0.012
Organizational Policy Support	0.183	0.008

### 3.6 Reliability analysis

Reliability coefficients calculated using the total sample are presented in Table 7. Across the six subscales, Cronbach’s α values ranged from 0.662 to 0.946, McDonald’s ω values ranged from 0.671 to 0.948, and Spearman–Brown split-half reliability coefficients ranged from 0.591 to 0.967. Mental Health, Physical Activity, Interpersonal Support, Built Environment Support, and Organizational Policy Support showed acceptable to excellent internal consistency, whereas the Diet and Nutrition subscale showed comparatively lower reliability (Cronbach’s α = 0.662, McDonald’s ω = 0.671, and split-half reliability = 0.591). Accordingly, internal consistency was stronger for the other five subscales than for Diet and Nutrition; the latter warrants further evaluation in an independent sample.

**Table 7:** Internal consistency reliability of the six MASE-HC subscales.

Subscale	Items	Cronbach’s α (*n* = 412)	McDonald’s ω (*n* = 412)	Spearman–Brown Split‑half reliability (*n* = 412)
Mental Health	6	0.881	0.894	0.877
Physical Activity	4	0.770	0.785	0.711
Diet and Nutrition	3	0.662	0.671	0.591
Interpersonal Support	9	0.912	0.916	0.921
Built Environment Support	8	0.922	0.924	0.947
Organizational Policy Support	9	0.946	0.948	0.967

Reliability coefficients were calculated using the total sample (*n* = 412). McDonald’s ω denotes one-factor omega total but is labeled simply as McDonald’s ω in the table. Split-half coefficients were based on an odd–even split defined by the prespecified item order and were corrected using the Spearman–Brown formula.

The total 39-item set also showed high internal consistency (Cronbach’s α = 0.941, McDonald’s ω = 0.949, and split-half reliability = 0.968), although interpretation of the MASE-HC should primarily focus on the six subscale scores. These findings indicate that internal consistency was generally satisfactory, while the Diet and Nutrition subscale may require further refinement.

## 4 Discussion

The final 39-item MASE-HC comprises six correlated first-order factors. The refined CFA model provided preliminary structural support, with TLI marginally below 0.90 and four post-hoc within-factor residual covariances. The Fornell–Larcker criterion was satisfied for all six factors; however, this single criterion should not be interpreted as comprehensive proof of discriminant validity because discriminant validity is not adequately represented by a binary cutoff alone (55). Discriminant validity was supported across the six factors, whereas convergent validity and internal consistency were weaker for Diet and Nutrition. The evidence is therefore initial rather than definitive and requires independent replication.

Community health assessment is best understood as a comprehensive, community-engaged process that integrates needs, assets, determinants, and resources to guide priority setting and action (9, 10). Community health assessment has received increasing attention worldwide and is important for healthy city development and population health promotion (7). Existing tools have assessed related but more specific aspects of community health, such as neighborhood environment scale (13), the Community Efficacy for Non-Communicable Disease Management (COEN) scale (14), the multi-dimensional Health Resilience Scale (HRS) (15), and the Healthy Lives 5 Axis Scale (HL-5) (16). These instruments provide useful measures for particular constructs or populations. In contrast, the MASE-HC was designed to integrate individual, interpersonal, environmental, and organizational-policy domains within a single social–ecological framework. More specifically, the MASE-HC was conceptualized as a multidimensional resident-reported instrument that characterizes individual health-related characteristics together with residents’ perceptions of interpersonal, built-environmental, and organizational-policy support. It was not intended to represent a single objective community-level trait. This structure may be useful for describing how residents perceive both their own health-related behaviors and the broader community supports that shape those behaviors. Nevertheless, the MASE-HC should be viewed as a complementary assessment tool rather than a replacement for existing instruments targeting specific constructs.

The scale development process followed standard procedures for item generation, expert review, item analysis, and factor validation. The initial item pool was constructed from the social–ecological model and relevant community health assessment literature, then refined through Delphi consultation, content validity assessment, cognitive interviews, and empirical item screening. This process helped ensure that the retained items had both theoretical relevance and acceptable statistical performance. EFA ultimately supported a six-factor structure comprising Mental Health, Physical Activity, Diet and Nutrition, Interpersonal Support, Built Environment Support, and Organizational Policy Support, which together explained 61.411% of the variance. The first three factors were conceptually grouped within the broader Individual Health and Behavior domain, whereas Interpersonal Support, Built Environment Support, and Organizational Policy Support corresponded to the remaining three broad domains. These broad domains were retained as conceptual groupings rather than modeled as higher-order latent factors. The emergence of three distinct individual-level factors suggests that psychological health, physical activity, and dietary behavior represent related but empirically distinguishable components within the Individual Health and Behavior domain.

The refined CFA model showed generally acceptable fit, with χ^2^/df = 1.741, RMSEA = 0.060, SRMR = 0.0649, CFI = 0.906, and IFI = 0.907; TLI = 0.898 was marginally below the conventional 0.90 reference value. Four within-factor residual covariances were added *post hoc* on the basis of modification indices and plausible item-content overlap. This localized respecification and the modest CFA subsample (*n* = 206) limit claims of confirmation. Replication in a larger independent sample is needed to determine whether the six-factor structure can be reproduced without post-hoc modification (46, 47).

Convergent-validity evidence varied across factors. Mental Health, Interpersonal Support, Built Environment Support, and Organizational Policy Support met the conventional CR and AVE criteria. Physical Activity had adequate composite reliability (CR = 0.791), while its AVE was marginally below 0.50 (AVE = 0.496). Diet and Nutrition showed the weakest evidence (CR = 0.674; AVE = 0.417), consistent with its lower α (0.662), McDonald’s ω (0.671), and split-half reliability (0.591). Its three-item length may contribute to lower internal-consistency estimates because such coefficients are influenced by test length (53, 56, 57). Short length, however, does not by itself explain away the combined CR, AVE, and reliability weaknesses. The subscale should therefore be treated as provisional and refined by revising current items or adding theoretically relevant dietary indicators in future studies. Discriminant validity was supported across all six factors: the highest correlation, between Interpersonal Support and Built Environment Support (*r* = 0.658), remained below $\sqrt{\mathrm{AVE}}$ for both factors (0.722 and 0.727).

The four broad domains provide a pragmatic conceptual mapping to the social–ecological model rather than a set of higher-order latent factors. A recent concept analysis similarly characterized healthy living as a multidimensional, life-course process spanning behavioral–physical, cognitive–psychological, social–relational, and contextual–structural dimensions. This contemporary framework supports the breadth of the MASE-HC conceptually, but it does not constitute empirical validation of the scale (19). Individual Health and Behavior covers the three individual-level factors—Mental Health, Physical Activity, and Diet and Nutrition—whereas Interpersonal Support reflects close social relationships and their influence on health behavior. Built Environment Support captures selected physical and resource features of the community environment, not the full community level. Organizational Policy Support combines residents’ perceptions of locally delivered organizational support, health services, and visible health-promoting policies; it does not reproduce the organizational and public-policy levels one-to-one. Because all contextual items are individual perceptions, the present data do not establish objective community performance, score aggregation, ranking, or between-community comparison.

The hypothesis-based external construct validity results should be interpreted cautiously. Rather than treating self-rated health as a criterion measure, the present study used it as a hypothesis-based external construct because no established gold-standard measure exists for the multidimensional construct assessed by the MASE-HC. Self-rated health is commonly interpreted as an integrative subjective appraisal of health, but it remains a broad single-item construct (51, 52). Self-rated health was positively associated with five of the six subscales. The strongest association was observed for Mental Health (*r*ₛ = 0.340), followed by Interpersonal Support, Organizational Policy Support, Built Environment Support, and Physical Activity, whereas Diet and Nutrition was not significantly associated with self-rated health. Overall, the correlations were weak to moderate, providing only limited supplementary evidence of external construct validity. This pattern is plausible because self-rated health primarily reflects an individual’s global perception of health and does not directly represent contextual constructs such as environmental or organizational-policy support. Accordingly, these associations should not be interpreted as criterion validation of the MASE-HC.

This study has several strengths. First, the MASE-HC was developed based on the social–ecological model and followed a structured scale development process, including literature review, Delphi consultation, content validity assessment, cognitive interviews, item analysis, EFA, and CFA. Second, the scale integrates individual, interpersonal, built environmental, and organizational-policy domains, providing a broader assessment framework than instruments focused on a single aspect of community health. Third, item analysis and EFA were conducted in one subsample, whereas CFA and subsequent construct-validity analyses were conducted in a separate confirmatory subsample, reducing direct reuse of the same observations across exploratory and confirmatory stages (22, 28). Fourth, the evaluation incorporated multiple complementary psychometric indices, including factor structure, composite reliability, AVE, Fornell–Larcker discriminant validity, Cronbach’s α, McDonald’s ω, and split-half reliability.

This study has several limitations. First, participants were recruited from a community health service center in Anhui Province using purposive sampling, which may introduce selection bias and limit the generalizability of the findings to other regions or populations (58). The sample was also relatively young (mean age approximately 34 years), predominantly male (63.6%), and comparatively highly educated, with more than half holding a bachelor’s degree or above. Therefore, older adults and residents with lower educational attainment were underrepresented. Second, the cross-sectional design did not allow assessment of predictive validity or causal relationships between community support and health outcomes. Third, although the sample was randomly divided into exploratory and confirmatory subsamples, this within-study split does not constitute fully independent external validation. In addition, the CFA subsample of 206 participants was modest for a 39-item six-factor model, and four residual covariances were introduced *post hoc*; therefore, replication of the refined structure in an independent, larger sample is essential. Fourth, the Diet and Nutrition subscale showed comparatively weak convergent validity and internal consistency, indicating a need for further item refinement and possibly expansion of this domain. Fifth, external construct evidence was limited to a single self-rated health item, which cannot fully represent the multidimensional individual and contextual components assessed by the MASE-HC. Finally, the current study assessed individual residents and did not evaluate measurement invariance across demographic groups or communities (59). Therefore, the present findings do not yet support use of the MASE-HC for direct community-level ranking or comparison.

Despite these limitations, the MASE-HC provides a theoretically grounded and empirically tested multidimensional resident-reported framework for characterizing individual health-related characteristics and perceived interpersonal, built-environmental, and organizational-policy supports relevant to community health promotion. Future studies should evaluate the scale psychometrically in different regions and populations, examine measurement invariance across demographic groups, and assess its responsiveness to community health interventions (59, 60). Independent multicenter studies with larger and more representative samples are particularly needed to confirm the six-factor structure, reassess the post-hoc residual correlations, and refine the Diet and Nutrition subscale.

Longitudinal research is also needed to determine whether changes in MASE-HC scores are associated with subsequent changes in health behaviors, self-rated health, or broader community health outcomes. Until these studies are completed, use of the MASE-HC is best framed as research-oriented and subscale specific, with especially cautious interpretation of Diet and Nutrition scores.

## 5 Conclusion

The MASE-HC provides a preliminary resident-reported framework for assessing individual health-related characteristics and perceived interpersonal, built-environmental, and organizational-policy supports relevant to community health promotion. The present study provided initial support for its six-factor structure, discriminant validity, and internal consistency, although the Diet and Nutrition subscale showed comparatively weaker convergent validity and reliability. Further item refinement and independent validation in more representative populations are required before routine application or community-level comparison.

## Data Availability

The raw data supporting the conclusions of this article will be made available by the authors, without undue reservation.

## References

[1] Stokols D. Translating social ecological theory into guidelines for community health promotion. Am J HEALTH Promot. (1996) 10:282–98. doi: 10.4278/0890-1171-10.4.282, 10159709

[2] Stokols D. Establishing and maintaining healthy environments. Toward a social ecology of health promotion. Am Psychol. (1992) 47:6–22. doi: 10.1037//0003-066x.47.1.6, 1539925

[3] Golden SD Earp JAL. Social ecological approaches to individuals and their contexts: twenty years of health education & behavior health promotion interventions. Health Educ Behav. (2012) 39:364–72. doi: 10.1177/1090198111418634, 22267868

[4] Sallis JF Owen N Fisher EB. Ecological models of health behavior. In: Glanz K, Rimer BK, Viswanath K, editors. Health Behavior: Theory, Research, and Practice. 5th ed. San Francisco, CA: Jossey-Bass (2015), p. 43–64.

[5] Winett RA. A framework for health promotion and disease prevention programs. Am Psychol. (1995) 50:341–50. doi: 10.1037//0003-066x.50.5.341, 7762887

[6] McLeroy KR Bibeau D Steckler A Glanz K. An ecological perspective on health promotion programs. Health Educ Q. (1988) 15:351–77. doi: 10.1177/109019818801500401, 3068205

[7] Irani P Bohn C Halasan C Landen M McCusker D. Community health assessment: driving the need for current, easily accessible population health data. J Public Health Manag Pract. (2006) 12:113–8. doi: 10.1097/00124784-200603000-00002 16479224

[8] Centers for Disease Control and Prevention. (2026) Community Health Assessments and Improvement Plans. Public Health Infrastructure Center. Available online at: https://www.cdc.gov/infrastructure/public-health-systems-emerging-practices/community-health-assessments-and-improvement-plans.html (Accessed September 1, 2026)

[9] Pazzaglia C Camedda C Ugenti NV Trentin A Scalorbi S Longobucco Y. Community health assessment tools adoptable in nursing practice: a scoping review. Int J Environ Res Public Health. (2023) 20:1667. doi: 10.3390/ijerph20031667, 36767035 PMC9914332

[10] Ravaghi H Guisset AL Elfeky S Nasir N Khani S Ahmadnezhad E . A scoping review of community health needs and assets assessment: concepts, rationale, tools and uses. BMC Health Serv Res. (2023) 23:44. doi: 10.1186/s12913-022-08983-3, 36650529 PMC9847055

[11] Zhao J Chen Y Zhang P. Socio-ecological factors in building a healthy community: a comparative study of healthy community assessment standards. Buildings. (2024) 14:2634. doi: 10.3390/buildings14092634

[12] Raudenbush SW Sampson RJ. Ecometrics: toward a science of assessing ecological settings, with application to the systematic social observation of neighborhoods. Sociol Methodol. (1999) 29:1–41. doi: 10.1111/0081-1750.00059

[13] Mujahid MS Diez Roux AV Morenoff JD Raghunathan T. Assessing the measurement properties of neighborhood scales: from psychometrics to ecometrics. Am J Epidemiol. (2007) 165:858–67. doi: 10.1093/aje/kwm040, 17329713

[14] Xiong S Zhu G Malhotra R Chen X Gong E Wang Z . Community efficacy for non-communicable disease management (COEN): conceptualization and measurement. PLOS Global Public Health. (2024) 4:e0003549. doi: 10.1371/journal.pgph.0003549, 39141640 PMC11324141

[15] Ge L Yip WF Li R Chua ESS Ho MHR Ho AHY . Development and validation of the multi-dimensional health resilience scale for community-dwelling adults. Front Public Health. (2025) 13:1452738. doi: 10.3389/fpubh.2025.1452738, 40013045 PMC11864133

[16] White H Gonzalez P Shashar S Silver Z Scott H Novack V. Healthy lives 5 axis scale for individuals with complex health and social needs: assessment of validity and reliability. BMC Health Serv Res. (2025) 25:880. doi: 10.1186/s12913-025-13069-x, 40598501 PMC12219705

[17] Papakosta-Gaki E Zissi A Tsounis A Kyritsakas E Ploukou S Sarafis P . Development and validation of the primary care needs assessment (PCNA) questionnaire: a participatory multidimensional approach to identifying health needs. HealthCare. (2026) 14:1302. doi: 10.3390/healthcare14101302, 42194392 PMC13206681

[18] Dehghani E Karimi K Sokke HGM Khadembashiri MA Ghavidel F Memari AH. Healthy lifestyle scales to assess general and clinical population: a systematic review with narrative synthesis. Lifestyle Med. (2024) 5:e104. doi: 10.1002/lim2.104

[19] Trujillo-Cáceres SJ Roa-Díaz ZM Figueroa CP Hernández Vargas JA Rojas LZ Aceros Lora AM . Healthy living concepts and definitions: a systematic review with concept analysis proposing a four-dimensional model. Maturitas. (2026) 210:108976. doi: 10.1016/j.maturitas.2026.108976 42167064

[20] DeVellis RF. Scale Development: Theory and Applications, vol. 26 3rd ed. Thousand Oaks, CA: SAGE Publications. (2012).

[21] Boateng GO Neilands TB Frongillo EA Melgar-Quiñonez HR Young SL. Best practices for developing and validating scales for health, social, and behavioral research: a primer. Front Public Health. (2018) 6:149. doi: 10.3389/fpubh.2018.00149, 29942800 PMC6004510

[22] Stefana A Damiani S Granziol U Provenzani U Solmi M Youngstrom EA . Psychological, psychiatric, and behavioral sciences measurement scales: best practice guidelines for their development and validation. Front Psychol. (2024) 15:1494261. doi: 10.3389/fpsyg.2024.1494261, 39916786 PMC11798685

[23] Hinkin TR. A brief tutorial on the development of measures for use in survey questionnaires. Organ Res Methods. (1998) 1:104–21. doi: 10.1177/109442819800100106

[24] Jünger S Payne SA Brine J Radbruch L Brearley SG. Guidance on conducting and REporting DElphi studies (CREDES) in palliative care: recommendations based on a methodological systematic review. Palliat Med. (2017) 31:684–706. doi: 10.1177/0269216317690685, 28190381

[25] Polit DF Beck CT. The content validity index: are you sure you know what’s being reported? Critique and recommendations. Res Nurs Health. (2006) 29:489–97. doi: 10.1002/nur.20147, 16977646

[26] Terwee CB Prinsen CAC Chiarotto A Westerman MJ Patrick DL Alonso J . COSMIN methodology for evaluating the content validity of patient-reported outcome measures: a delphi study. Qual Life Res. (2018) 27:1159–70. doi: 10.1007/s11136-018-1829-0, 29550964 PMC5891557

[27] Beatty PC Willis GB. Research synthesis: the practice of cognitive interviewing. Public Opin Q. (2007) 71:287–311. doi: 10.1093/poq/nfm006

[28] Fokkema M Greiff S. How performing PCA and CFA on the same data equals trouble: overfitting in the assessment of internal structure and some editorial thoughts on it. Eur J Psychol Assess. (2017) 33:399–402. doi: 10.1027/1015-5759/a000460

[29] MacCallum RC Widaman KF Zhang S Hong S. Sample size in factor analysis. Psychol Methods. (1999) 4:84–99. doi: 10.1037/1082-989X.4.1.84 26822184

[30] Lorenzo-Seva U Ferrando PJ. Determining sample size requirements in EFA solutions: a simple empirical proposal. Multivar Behav Res. (2024) 59:899–912. doi: 10.1080/00273171.2024.2342324, 38717588

[31] Büyüköztürk Ş Çokluk Bökeoğlu Ö. Discriminant function analysis: concept and application. Egit Arastirmalari Eurasian J Educ Res. (2008) 8:73–92.

[32] Kelley TL. The selection of upper and lower groups for the validation of test items. J Educ Psychol. (1939) 30:17–24. doi: 10.1037/h0057123

[33] Zhang D Zheng H Zeng Y Yu X Zhao Y Gan Y . Psychometric properties of the chinese version of the quiet quitting scale. BMC Nurs. (2025) 24:270. doi: 10.1186/s12912-025-02921-4, 40069793 PMC11900426

[34] Norris M Lecavalier L. Evaluating the use of exploratory factor analysis in developmental disability psychological research. J Autism Dev Disord. (2010) 40:8–20. doi: 10.1007/s10803-009-0816-2, 19609833

[35] Watkins MW. Exploratory factor analysis: a guide to best practice. J Black Psychol. (2018) 44:219–46. doi: 10.1177/0095798418771807

[36] Goretzko D Pham TTH Bühner M. Exploratory factor analysis: current use, methodological developments and recommendations for good practice. Curr Psychol. (2021) 40:3510–21. doi: 10.1007/s12144-019-00300-2

[37] Son YJ Baek KH Won MH Hong HC. Development and psychometric testing of the self-care scale for patients with chronic atrial fibrillation (AF-SCS-10). Eur J Cardiovasc Nurs. (2020) 19:619–28. doi: 10.1177/1474515120920472, 32340477

[38] Schreiber JB. Issues and recommendations for exploratory factor analysis and principal component analysis. Res Soc Adm Pharm. (2021) 17:1004–11. doi: 10.1016/j.sapharm.2020.07.027 33162380

[39] Horn JL. A rationale and test for the number of factors in factor analysis. Psychometrika. (1965) 30:179–85. doi: 10.1007/BF02289447, 14306381

[40] Timmerman ME Lorenzo-Seva U. Dimensionality assessment of ordered polytomous items with parallel analysis. Psychol Methods. (2011) 16:209–20. doi: 10.1037/a0023353, 21500916

[41] Wang YY. Development and Evaluation of Medical Scales: Theory, Methods, and Practical Examples. Beijing: Peking University Medical Press (2020).

[42] Rhemtulla M Brosseau-Liard PÉ Savalei V. When can categorical variables be treated as continuous? A comparison of robust continuous and categorical SEM estimation methods under suboptimal conditions. Psychol Methods. (2012) 17:354–73. doi: 10.1037/a0029315, 22799625

[43] Li CH. Confirmatory factor analysis with ordinal data: comparing robust maximum likelihood and diagonally weighted least squares. Behav Res Methods. (2016) 48:936–49. doi: 10.3758/s13428-015-0619-7, 26174714

[44] Groskurth K Bluemke M Lechner CM. Why we need to abandon fixed cutoffs for goodness-of-fit indices: an extensive simulation and possible solutions. Behav Res Methods. (2024) 56:3891–914. doi: 10.3758/s13428-023-02193-3, 37640961 PMC11133148

[45] Hu L t Bentler PM. Cutoff criteria for fit indexes in covariance structure analysis: conventional criteria versus new alternatives. Struct Equ Model Multidiscip J. (1999) 6:1–55. doi: 10.1080/10705519909540118

[46] MacCallum RC Roznowski M Necowitz LB. Model modifications in covariance structure analysis: the problem of capitalization on chance. Psychol Bull. (1992) 111:490–504. doi: 10.1037/0033-2909.111.3.490, 16250105

[47] Saris WE Satorra A van der Veld WM. Testing structural equation models or detection of misspecifications? Struct Equ Model Multidiscip J. (2009) 16:561–82. doi: 10.1080/10705510903203433

[48] Minglong W. The Practice of Questionnaire Statistical Analysis: Operation and Application of SPSS. Chongqing: Chongqing University Press (2011).

[49] Fornell C Larcker DF. Evaluating structural equation models with unobservable variables and measurement error. J Mark Res. (1981) 18:39–50. doi: 10.1177/002224378101800104

[50] Henseler J Ringle CM Sarstedt M. A new criterion for assessing discriminant validity in variance-based structural equation modeling. J Acad Mark Sci. (2015) 43:115–35. doi: 10.1007/s11747-014-0403-8

[51] Idler EL Benyamini Y. Self-rated health and mortality: a review of twenty-seven community studies. J Health Soc Behav. (1997) 38:21–37. doi: 10.2307/2955359, 9097506

[52] Jylhä M. What is self-rated health and why does it predict mortality? Towards a unified conceptual model. Soc Sci Med. (2009) 69:307–16. doi: 10.1016/j.socscimed.2009.05.013, 19520474

[53] Dunn TJ Baguley T Brunsden V. From alpha to omega: a practical solution to the pervasive problem of internal consistency estimation. Br J Psychol. (2014) 105:399–412. doi: 10.1111/bjop.12046, 24844115

[54] Akoglu H. User’s guide to correlation coefficients. Turk J Emerg Med. (2018) 18:91–3. doi: 10.1016/j.tjem.2018.08.001, 30191186 PMC6107969

[55] Rönkkö M Cho E. An updated guideline for assessing discriminant validity. Organ Res Methods. (2022) 25:6–14. doi: 10.1177/1094428120968614

[56] McNeish D. Thanks coefficient alpha, we’ll take it from here. Psychol Methods. (2018) 23:412–33. doi: 10.1037/met0000144, 28557467

[57] Hayes AF Coutts JJ. Use omega rather than cronbach’s alpha for estimating reliability. But…. Commun Methods Meas. (2020) 14:1–24. doi: 10.1080/19312458.2020.1718629

[58] Bornstein MH Jager J Putnick DL. Sampling in developmental science: situations, shortcomings, solutions, and standards. Dev Rev: DR. (2013) 33:357–70. doi: 10.1016/j.dr.2013.08.003, 25580049 PMC4286359

[59] Putnick DL Bornstein MH. Measurement invariance conventions and reporting: the state of the art and future directions for psychological research. Dev Rev: DR. (2016) 41:71–90. doi: 10.1016/j.dr.2016.06.004, 27942093 PMC5145197

[60] Mokkink LB Terwee CB Patrick DL Alonso J Stratford PW Knol DL . The COSMIN study reached international consensus on taxonomy, terminology, and definitions of measurement properties for health-related patient-reported outcomes. J Clin Epidemiol. (2010) 63:737–45. doi: 10.1016/j.jclinepi.2010.02.006, 20494804

