# Supplementary material for: Measuring residents’ perceptions of social-ecological healthy communities: development and initial psychometric evaluation of a multidimensional scale
Source: Front Public Health. 2026 Oct 7;14:1957359. doi: 10.3389/fpubh.2026.1957359 (PMC13683629; doi:10.3389/fpubh.2026.1957359)
Supplement: Supplementary file 1 [file Supplementary_file_1.DOCX]

**Supplementary File 1. Sequential Item Analysis of the MASE-HC in Subsample 1 (*n* = 206)**

**Panel A. Critical-ratio analysis**

| **Item** | **Critical ratio** | ***P*** | **Decision at this stage** |
| --- | --- | --- | --- |
| A1 | 1.654 | 0.101 | Delete |
| A2 | 2.648 | 0.009 | Delete |
| A3 | 3.151 | 0.002 | Retain |
| A4 | 10.454 | <0.001 | Retain |
| A5 | 11.030 | <0.001 | Retain |
| A6 | 10.699 | <0.001 | Retain |
| A7 | 7.497 | <0.001 | Retain |
| A8 | 6.262 | <0.001 | Retain |
| A9 | 9.489 | <0.001 | Retain |
| A10 | 3.792 | <0.001 | Retain |
| A11 | 4.707 | <0.001 | Retain |
| A12 | 3.619 | <0.001 | Retain |
| A13 | 3.785 | <0.001 | Retain |
| A14 | 1.072 | 0.286 | Delete |
| A15 | 4.397 | <0.001 | Retain |
| A16 | 4.750 | <0.001 | Retain |
| A17 | 5.285 | <0.001 | Retain |
| A18 | 4.183 | <0.001 | Retain |
| A19 | -1.972 | 0.051 | Delete |
| A20 | 0.685 | 0.494 | Delete |
| A21 | 3.499 | 0.001 | Retain |
| A22 | 4.913 | <0.001 | Retain |
| A23 | 0.273 | 0.786 | Delete |
| A24 | 2.273 | 0.025 | Delete |
| A25 | 6.654 | <0.001 | Retain |
| A26 | 1.856 | 0.066 | Delete |
| A27 | 6.674 | <0.001 | Retain |
| A28 | 1.958 | 0.053 | Delete |
| A29 | 1.712 | 0.090 | Delete |
| B1 | 9.955 | <0.001 | Retain |
| B2 | 9.505 | <0.001 | Retain |
| B3 | 9.550 | <0.001 | Retain |
| B4 | 9.028 | <0.001 | Retain |
| B5 | 9.125 | <0.001 | Retain |
| B6 | 9.190 | <0.001 | Retain |
| B7 | 9.897 | <0.001 | Retain |
| B8 | 7.782 | <0.001 | Retain |
| B9 | 9.874 | <0.001 | Retain |
| B10 | 9.057 | <0.001 | Retain |
| B11 | 7.838 | <0.001 | Retain |
| B12 | 5.958 | <0.001 | Retain |
| C1 | 7.795 | <0.001 | Retain |
| C2 | 10.541 | <0.001 | Retain |
| C3 | 10.212 | <0.001 | Retain |
| C4 | 10.655 | <0.001 | Retain |
| C5 | 9.517 | <0.001 | Retain |
| C6 | 10.399 | <0.001 | Retain |
| C7 | 11.785 | <0.001 | Retain |
| C8 | 10.010 | <0.001 | Retain |
| C9 | 7.646 | <0.001 | Retain |
| C10 | 9.578 | <0.001 | Retain |
| C11 | 6.101 | <0.001 | Retain |
| D1 | 10.377 | <0.001 | Retain |
| D2 | 10.253 | <0.001 | Retain |
| D3 | 10.379 | <0.001 | Retain |
| D4 | 9.224 | <0.001 | Retain |
| D5 | 9.477 | <0.001 | Retain |
| D6 | 10.564 | <0.001 | Retain |
| D7 | 9.475 | <0.001 | Retain |
| D8 | 8.024 | <0.001 | Retain |
| D9 | 6.775 | <0.001 | Retain |

**Panel B. Corrected item–domain correlations**

| **Item** | **Corrected item–domain correlation** | **Decision at this stage** |
| --- | --- | --- |
| A3 | 0.207** | Delete |
| A4 | 0.654** | Retain |
| A5 | 0.671** | Retain |
| A6 | 0.606** | Retain |
| A7 | 0.509** | Retain |
| A8 | 0.481** | Retain |
| A9 | 0.595** | Retain |
| A10 | 0.466** | Retain |
| A11 | 0.560** | Retain |
| A12 | 0.449** | Retain |
| A13 | 0.462** | Retain |
| A15 | 0.465** | Retain |
| A16 | 0.420** | Retain |
| A17 | 0.467** | Retain |
| A18 | 0.422** | Retain |
| A21 | 0.345** | Retain |
| A22 | 0.378** | Retain |
| A25 | 0.507** | Retain |
| A27 | 0.484** | Retain |
| B1 | 0.784** | Retain |
| B2 | 0.780** | Retain |
| B3 | 0.802** | Retain |
| B4 | 0.756** | Retain |
| B5 | 0.593** | Retain |
| B6 | 0.684** | Retain |
| B7 | 0.776** | Retain |
| B8 | 0.744** | Retain |
| B9 | 0.759** | Retain |
| B10 | 0.798** | Retain |
| B11 | 0.635** | Retain |
| B12 | 0.607** | Retain |
| C1 | 0.734** | Retain |
| C2 | 0.855** | Retain |
| C3 | 0.809** | Retain |
| C4 | 0.838** | Retain |
| C5 | 0.855** | Retain |
| C6 | 0.798** | Retain |
| C7 | 0.753** | Retain |
| C8 | 0.770** | Retain |
| C9 | 0.682** | Retain |
| C10 | 0.718** | Retain |
| C11 | 0.625** | Retain |
| D1 | 0.754** | Retain |
| D2 | 0.833** | Retain |
| D3 | 0.735** | Retain |
| D4 | 0.852** | Retain |
| D5 | 0.878** | Retain |
| D6 | 0.884** | Retain |
| D7 | 0.865** | Retain |
| D8 | 0.812** | Retain |
| D9 | 0.798** | Retain |

**Panel C. Cronbach’s α if item deleted**

| Item | Cronbach’s α if item deleted | Decision at this stage |
| --- | --- | --- |
| A4 | 0.947 | Retain |
| A5 | 0.947 | Retain |
| A6 | 0.947 | Retain |
| A7 | 0.947 | Retain |
| A8 | 0.948 | Retain |
| A9 | 0.947 | Retain |
| A10 | 0.949 | Retain |
| A11 | 0.949 | Retain |
| A12 | 0.949 | Retain |
| A13 | 0.950 | Retain |
| A15 | 0.949 | Retain |
| A16 | 0.949 | Retain |
| A17 | 0.949 | Retain |
| A18 | 0.949 | Retain |
| A21 | 0.949 | Retain |
| A22 | 0.949 | Retain |
| A25 | 0.948 | Retain |
| A27 | 0.948 | Retain |
| B1 | 0.947 | Retain |
| B2 | 0.947 | Retain |
| B3 | 0.947 | Retain |
| B4 | 0.947 | Retain |
| B5 | 0.947 | Retain |
| B6 | 0.947 | Retain |
| B7 | 0.947 | Retain |
| B8 | 0.947 | Retain |
| B9 | 0.947 | Retain |
| B10 | 0.947 | Retain |
| B11 | 0.948 | Retain |
| B12 | 0.948 | Retain |
| C1 | 0.947 | Retain |
| C2 | 0.946 | Retain |
| C3 | 0.947 | Retain |
| C4 | 0.946 | Retain |
| C5 | 0.947 | Retain |
| C6 | 0.947 | Retain |
| C7 | 0.946 | Retain |
| C8 | 0.947 | Retain |
| C9 | 0.947 | Retain |
| C10 | 0.947 | Retain |
| C11 | 0.948 | Retain |
| D1 | 0.946 | Retain |
| D2 | 0.946 | Retain |
| D3 | 0.947 | Retain |
| D4 | 0.946 | Retain |
| D5 | 0.947 | Retain |
| D6 | 0.946 | Retain |
| D7 | 0.947 | Retain |
| D8 | 0.947 | Retain |
| D9 | 0.948 | Retain |

*Note.* ** P < 0.01. A, B, C, and D denote the four prespecified content domains: Individual Health and Behavior, Interpersonal Support, Built Environment Support, and Organizational Policy Support. Item analysis was conducted sequentially in Subsample 1 (n = 206). All 61 field-test items were first examined using the critical-ratio method. The 51 items retained at this stage were subsequently evaluated using corrected item–domain correlations. After A3 was removed, the remaining 50 items were examined using Cronbach’s α if item deleted for the retained item set; this statistic was used only as an item-screening indicator. Consequently, the number of items differed across the three panels.
