# Supplementary material for: Measuring residents’ perceptions of social-ecological healthy communities: development and initial psychometric evaluation of a multidimensional scale
Source: Front Public Health. 2026 Oct 7;14:1957359. doi: 10.3389/fpubh.2026.1957359 (PMC13683629; doi:10.3389/fpubh.2026.1957359)
Supplement: Supplementary file 2 [file Supplementary_file_2.DOCX]

g

Eigenva lue

Common−factor e i enva lue

5	6	7	8

Factor

12	13	14	15	16	17	18	19	20

Observed data

Simulated random data (95th percentile)

1	2	3	4	5	6	7	8	9	10	11

Factor 6: Observed 0.845	Random 0.776

Factor 7: Observed 0.674	Random 0.716

15

10

1.8

1.6

1.4

1.2

1.0

0.8

0.6

0.4

Factor number

**Decision boundary**

5

0
