# Supplementary material for: Measuring residents’ perceptions of social-ecological healthy communities: development and initial psychometric evaluation of a multidimensional scale
Source: Front Public Health. 2026 Oct 7;14:1957359. doi: 10.3389/fpubh.2026.1957359 (PMC13683629; doi:10.3389/fpubh.2026.1957359)
Supplement: Supplementary file 3 [file Supplementary_file_3.DOCX]

**Supplementary File 3. Final exploratory factor analysis pattern matrix for the 39-item MASE-HC (*n* = 206)**

| **Item** | **Factor** | | | | | |
| --- | --- | --- | --- | --- | --- | --- |
|  | **1** | **2** | **3** | **4** | **5** | **6** |
| A4 | -0.792 |  |  |  |  |  |
| A5 | -0.927 |  |  |  |  |  |
| A6 | -0.811 |  |  |  |  |  |
| A7 | -0.613 |  |  |  |  |  |
| A8 | -0.568 |  |  |  |  |  |
| A9 | -0.722 |  |  |  |  |  |
| A10 |  | 0.765 |  |  |  |  |
| A11 |  | 0.692 |  |  |  |  |
| A12 |  | 0.703 |  |  |  |  |
| A13 |  | 0.420 |  |  |  |  |
| A16 |  |  | 0.614 |  |  |  |
| A17 |  |  | 0.524 |  |  |  |
| A18 |  |  | 0.672 |  |  |  |
| B1 |  |  |  | 0.547 |  |  |
| B2 |  |  |  | 0.604 |  |  |
| B3 |  |  |  | 0.704 |  |  |
| B4 |  |  |  | 0.663 |  |  |
| B6 |  |  |  | 0.476 |  |  |
| B7 |  |  |  | 0.685 |  |  |
| B8 |  |  |  | 0.799 |  |  |
| B9 |  |  |  | 0.653 |  |  |
| B10 |  |  |  | 0.605 |  |  |
| C1 |  |  |  |  | 0.629 |  |
| C2 |  |  |  |  | 0.833 |  |
| C3 |  |  |  |  | 0.798 |  |
| C4 |  |  |  |  | 0.840 |  |
| C5 |  |  |  |  | 0.852 |  |
| C6 |  |  |  |  | 0.750 |  |
| C7 |  |  |  |  | 0.488 |  |
| C8 |  |  |  |  | 0.580 |  |
| D1 |  |  |  |  |  | -0.535 |
| D2 |  |  |  |  |  | -0.792 |
| D3 |  |  |  |  |  | -0.581 |
| D4 |  |  |  |  |  | -0.766 |
| D5 |  |  |  |  |  | -0.894 |
| D6 |  |  |  |  |  | -0.872 |
| D7 |  |  |  |  |  | -0.812 |
| D8 |  |  |  |  |  | -0.699 |
| D9 |  |  |  |  |  | -0.742 |

Note. A, B, C, and D denote the four prespecified content domains: Individual Health and Behavior, Interpersonal Support, Built Environment Support, and Organizational Policy Support. Factors 1–6 correspond to Mental Health, Physical Activity, Diet and Nutrition, Interpersonal Support, Built Environment Support, and Organizational Policy Support, respectively. Factor loadings shown are from the final six-factor exploratory factor analysis using principal axis factoring with Direct Oblimin rotation.
