# Supplementary material for: Measuring residents’ perceptions of social-ecological healthy communities: development and initial psychometric evaluation of a multidimensional scale
Source: Front Public Health. 2026 Oct 7;14:1957359. doi: 10.3389/fpubh.2026.1957359 (PMC13683629; doi:10.3389/fpubh.2026.1957359)
Supplement: Supplementary file 4 [file Supplementary_file_4.DOCX]

**Supplementary File 4. The 39-item MASE-HC scale**

| **Subscale** | **Item ID** | **Item** |
| --- | --- | --- |
| **Mental Health** | A4 | I feel that I live a happy life. |
|  | A5 | I feel that my life is meaningful. |
|  | A6 | I can concentrate when thinking or doing things. |
|  | A7 | I can accept my physical appearance. |
|  | A8 | I often experience negative feelings, such as low mood, anxiety, or depression. |
|  | A9 | Overall, I am satisfied with myself. |
| **Physical Activity** | A10 | How often do I engage in vigorous exercise that makes me sweat or breathe heavily (e.g., basketball, soccer, running, swimming, dancing, or rope skipping) for more than 30 minutes per day? |
|  | A11 | How often do I engage in moderate-intensity leisure-time physical activity (e.g., brisk walking, cycling at a usual pace, shuttlecock kicking, or tai chi) for more than 30 minutes per day? |
|  | A12 | How often do I engage in muscle-strengthening activities (e.g., push-ups, sit-ups, or dumbbell exercises) for more than 30 minutes per day? |
|  | A13 | How often do I walk (low-intensity activity, e.g., walking to or from work) for more than 30 minutes per day? |
| **Diet and Nutrition** | A16 | How often do I consume vegetables, excluding starchy vegetables such as potatoes, sweet potatoes, yams, taro, and pumpkin? |
|  | A17 | How often do I consume fresh fruit, excluding fruit juice or canned fruit? |
|  | A18 | How often do I consume high-quality protein foods (e.g., fish, poultry, eggs, soy products, dairy products)? |
| **Interpersonal Support** | B1 | People around me remind or advise me to pay attention to health check-ups and my general health. |
|  | B2 | If I become ill, someone will provide practical care or assistance. |
|  | B3 | In daily life, I feel cared for and supported. |
|  | B4 | When I encounter difficulties in life, I have someone I trust to talk to. |
|  | B6 | People around me support and encourage me to exercise regularly. |
|  | B7 | I often prepare or share healthy meals with people around me. |
|  | B8 | I believe that the dietary advice from people around me is reliable and helpful. |
|  | B9 | If I stay up late or keep an irregular schedule, people around me remind me to adjust my routine. |
|  | B10 | If needed, people around me would actively help me create a quiet and comfortable sleep environment (e.g., dimming the lights, maintaining an appropriate room temperature, or reducing noise). |
| **Built Environment Support** | C1 | Basic health services in my community (e.g., vaccinations, health check-ups, and diagnosis and treatment of common diseases) are easy to access. |
|  | C2 | Nearby green spaces, rest areas, or quiet spaces are easy to access (within a 15-minute walk). |
|  | C3 | The surrounding natural scenery or environment helps me relax. |
|  | C4 | Walking paths, running paths, or sports venues are easy to access (within a 15-minute walk). |
|  | C5 | The overall community environment supports walking or physical activity (e.g., level paths, clear signage, and well-maintained facilities). |
|  | C6 | Healthy foods (e.g., fresh fruits and vegetables, whole grains, high-quality protein foods, and minimally processed foods) are readily available in stores. |
|  | C7 | Healthy foods are reasonably priced and affordable. |
|  | C8 | My bedroom environment (layout and atmosphere) is comfortable and helps me fall asleep and relax. |
| **Organizational Policy Support** | D1 | I am aware of and can access information on government or organizational policies or services related to disease prevention and health promotion (e.g., free health check-ups for specific populations, health lectures, and contracted family doctor services). |
|  | D2 | I feel that my community, workplace, or school has adequately implemented mental health support (e.g., mental health education activities and lectures on stress management). |
|  | D3 | I believe that the government or relevant organizations have actively advocated increasing physical activity (e.g., health trail signage, exercise health education posters, and “10,000 steps a day” campaigns). |
|  | D4 | I feel that my community, workplace, or school has adequately implemented measures to promote physical activity (e.g., building new sports facilities and organizing weight management lectures). |
|  | D5 | I am aware of and can access information on government or organizational policies or services related to food safety and healthy diets (e.g., dietary guideline campaigns and the “three reductions” initiative: reducing salt, oil, and sugar). |
|  | D6 | I feel that my community, workplace, or school has performed well in implementing healthy diet-related measures (e.g., advocating healthy dietary concepts and disseminating knowledge about healthy eating). |
|  | D7 | I feel that my community, workplace, or school has taken concrete actions to promote healthy sleep schedules (e.g., organizing lectures on sleep science). |
|  | D8 | I believe that the government strictly enforces the smoking ban in public places. |
|  | D9 | I believe that the government has effectively implemented health education efforts on limiting alcohol consumption. |

Note: The final MASE-HC comprises six subscales: Mental Health, Physical Activity, Diet and Nutrition, Interpersonal Support, Built Environment Support, and Organizational Policy Support. Items are rated on a 5-point Likert scale. Negatively worded items are reverse-coded using 6 minus the raw score so that higher scores consistently indicate more favorable health-related characteristics or stronger perceived social-ecological support. Subscale scores are calculated separately, and a single overall score is not used as the primary interpretive score.
