# Supplementary material for: Measuring residents’ perceptions of social-ecological healthy communities: development and initial psychometric evaluation of a multidimensional scale
Source: Front Public Health. 2026 Oct 7;14:1957359. doi: 10.3389/fpubh.2026.1957359 (PMC13683629; doi:10.3389/fpubh.2026.1957359)
Supplement: Supplementary file 5 [file Supplementary_file_5.DOCX]

**Multidimensional Assessment Scale for Social-Ecological Healthy Communities**

School of Public Health

www.ahmu.edu.cn

ID: ______________________________

**Dear participant:**

Thank you for taking part in this survey. This survey is intended for adult community residents aged 18 years or older. The Multidimensional Assessment Scale for Social-Ecological Healthy Communities is a resident-reported instrument designed to characterize individual health-related characteristics and residents’ perceptions of contextual supports relevant to social-ecological healthy communities.

Based on the social-ecological model, the scale covers four broad content domains: Individual Health and Behavior, Interpersonal Support, Built Environment Support, and Organizational Policy Support. It collects information on residents’ health-related behaviors and their perceptions of interpersonal, built-environmental, and organizational-policy support, providing a multidimensional description of social-ecological factors relevant to community health.

The data collected in this survey will be used only for scientific research. Your privacy will be protected, and your personal information will not be disclosed.

School of Public Health, Anhui Medical University

# I. Basic Information

Name: ____________________

Sex: ☐ Male ☐ Female

Date of birth: ______ year ______ month

Height (cm): __________

Weight (kg): __________

What is your highest level of education?

☐ Primary school or below	☐ Junior high school	☐ Senior high school	☐ Associate degree	☐ Bachelor’s degree	☐ Graduate degree

What is your occupation, or your occupation before retirement?

☐ Heads/managers of government agencies, Party and mass organizations, enterprises, or public institutions

☐ Professional and technical personnel

☐ Commercial or service personnel

☐ Agricultural, forestry, animal husbandry, fishery, or water-conservancy workers

☐ Homemaker	☐ Student	☐ Transport equipment operators and related personnel	☐ Other	☐ Unemployed

Which of the following best describes your type of work?

☐ Sedentary work (mostly sitting, such as office work, clerical work, or customer service)

☐ Standing work (requiring prolonged standing, such as sales, teaching, retail assistance, or nursing)

☐ Physically demanding work (requiring substantial physical activity, such as moving goods, delivery work, construction, or production-line work)

☐ Mixed work (a combination of sitting, standing, and walking, such as physicians, community workers, or service-sector workers)

☐ Other, please specify: ____________________

What is your marital status?

☐ Married	☐ Never married	☐ Cohabiting	☐ Separated	☐ Divorced or widowed

Including yourself, how many people currently live with you?

☐ 1 person	☐ 2 people	☐ 3 people	☐ 4 people	☐ 5 people	☐ 6 or more people

The figure below shows a 10-rung ladder representing the social classes of different families in China. A higher rung indicates a higher socioeconomic position. Rung 1 represents the lowest social class, and rung 10 represents the highest social class.

Which rung do you think best represents your position in society overall? ______ (enter a number)

How often do you visit a dentist?

☐ Every six months	☐ Once a year	☐ Once every two years	☐ Only when I have toothache or an oral health problem	☐ Never	☐ Do not know/Not sure

How much were your medical expenses in the past 3 months?

☐ Not applicable (no accidental injury or illness)	☐ Less than RMB 1,000	☐ RMB 1,000–3,000	☐ RMB 3,000–5,000	☐ RMB 5,000–10,000	☐ RMB 10,000–20,000	☐ RMB 20,000–30,000	☐ RMB 30,000–50,000	☐ More than RMB 50,000	☐ Do not know/Prefer not to answer

Overall, how would you rate your health?

☐ Very poor	☐ Poor	☐ Fair	☐ Good	☐ Very good

# II. Individual Health and Behavior

The following items ask about your experiences during the past month. Please choose the option that best reflects your situation.

| **Item ID** | **Item** | **Strongly disagree** | **Disagree** | **Somewhat agree** | **Agree** | **Strongly agree** |
| --- | --- | --- | --- | --- | --- | --- |
| A4 | I feel that I live a happy life. |  |  |  |  |  |
| A5 | I feel that my life is meaningful. |  |  |  |  |  |
| A6 | I can concentrate when thinking or doing things. |  |  |  |  |  |
| A7 | I can accept my physical appearance. |  |  |  |  |  |
| A8 | I often experience negative feelings, such as low mood, anxiety, or depression. |  |  |  |  |  |
| A9 | Overall, I am satisfied with myself. |  |  |  |  |  |

The following items ask about the frequency of physical activity during the past month.

| **Item ID** | **Item** | **Very rarely (0 days)** | **Rarely (1-2 days)** | **Occasionally (3-4 days)** | **Often (5-6 days)** | **Always (7 days)** |
| --- | --- | --- | --- | --- | --- | --- |
| A10 | How often do I engage in vigorous exercise that makes me sweat or breathe heavily (e.g., basketball, soccer, running, swimming, dancing, or rope skipping) for more than 30 minutes per day? |  |  |  |  |  |
| A11 | How often do I engage in moderate-intensity leisure-time physical activity (e.g., brisk walking, cycling at a usual pace, shuttlecock kicking, or tai chi) for more than 30 minutes per day? |  |  |  |  |  |
| A12 | How often do I engage in muscle-strengthening activities (e.g., push-ups, sit-ups, or dumbbell exercises) for more than 30 minutes per day? |  |  |  |  |  |
| A13 | How often do I walk (low-intensity activity, e.g., walking to or from work) for more than 30 minutes per day? |  |  |  |  |  |

The following items ask about the frequency of food intake during the past month.

| **Item ID** | **Item** | **Never (0 times)** | **Rarely (1-2 times/week)** | **Occasionally (3-4 times/week)** | **Often (5-6 times/week)** | **Daily** |
| --- | --- | --- | --- | --- | --- | --- |
| A16 | How often do I consume vegetables, excluding starchy vegetables such as potatoes, sweet potatoes, yams, taro, and pumpkin? |  |  |  |  |  |
| A17 | How often do I consume fresh fruit, excluding fruit juice or canned fruit? |  |  |  |  |  |
| A18 | How often do I consume high-quality protein foods (e.g., fish, poultry, eggs, soy products, dairy products)? |  |  |  |  |  |

# III. Interpersonal Support

The following items ask about your interactions, support, and health-related communication with people around you during the past month.

| **Item ID** | **Item** | **Strongly disagree** | **Disagree** | **Somewhat agree** | **Agree** | **Strongly agree** |
| --- | --- | --- | --- | --- | --- | --- |
| B1 | People around me remind or advise me to pay attention to health check-ups and my general health. |  |  |  |  |  |
| B2 | If I become ill, someone will provide practical care or assistance. |  |  |  |  |  |
| B3 | In daily life, I feel cared for and supported. |  |  |  |  |  |
| B4 | When I encounter difficulties in life, I have someone I trust to talk to. |  |  |  |  |  |
| B6 | People around me support and encourage me to exercise regularly. |  |  |  |  |  |
| B7 | I often prepare or share healthy meals with people around me. |  |  |  |  |  |
| B8 | I believe that the dietary advice from people around me is reliable and helpful. |  |  |  |  |  |
| B9 | If I stay up late or keep an irregular schedule, people around me remind me to adjust my routine. |  |  |  |  |  |
| B10 | If needed, people around me would actively help me create a quiet and comfortable sleep environment (e.g., dimming the lights, maintaining an appropriate room temperature, or reducing noise). |  |  |  |  |  |

# IV. Built Environment Support

The following items ask about your perceptions of how your residential environment supports health-related behaviors during the past month. In these items, “community” refers to the area within a 10- to 15-minute walk from your home.

| **Item ID** | **Item** | **Strongly disagree** | **Disagree** | **Somewhat agree** | **Agree** | **Strongly agree** |
| --- | --- | --- | --- | --- | --- | --- |
| C1 | Basic health services in my community (e.g., vaccinations, health check-ups, and diagnosis and treatment of common diseases) are easy to access. |  |  |  |  |  |
| C2 | Nearby green spaces, rest areas, or quiet spaces are easy to access (within a 15-minute walk). |  |  |  |  |  |
| C3 | The surrounding natural scenery or environment helps me relax. |  |  |  |  |  |
| C4 | Walking paths, running paths, or sports venues are easy to access (within a 15-minute walk). |  |  |  |  |  |
| C5 | The overall community environment supports walking or physical activity (e.g., level paths, clear signage, and well-maintained facilities). |  |  |  |  |  |
| C6 | Healthy foods (e.g., fresh fruits and vegetables, whole grains, high-quality protein foods, and minimally processed foods) are readily available in stores. |  |  |  |  |  |
| C7 | Healthy foods are reasonably priced and affordable. |  |  |  |  |  |
| C8 | My bedroom environment (layout and atmosphere) is comfortable and helps me fall asleep and relax. |  |  |  |  |  |

# V. Organizational Policy Support

The following items ask about your perceptions of health-related organizational and policy support during the past month. In these items, “organization” refers to the community, workplace, or school that you are currently involved with in your daily life.

| **Item ID** | **Item** | **Strongly disagree** | **Disagree** | **Somewhat agree** | **Agree** | **Strongly agree** |
| --- | --- | --- | --- | --- | --- | --- |
| D1 | I am aware of and can access information on government or organizational policies or services related to disease prevention and health promotion (e.g., free health check-ups for specific populations, health lectures, and contracted family doctor services). |  |  |  |  |  |
| D2 | I feel that my community, workplace, or school has adequately implemented mental health support (e.g., mental health education activities and lectures on stress management). |  |  |  |  |  |
| D3 | I believe that the government or relevant organizations have actively advocated increasing physical activity (e.g., health trail signage, exercise health education posters, and “10,000 steps a day” campaigns). |  |  |  |  |  |
| D4 | I feel that my community, workplace, or school has adequately implemented measures to promote physical activity (e.g., building new sports facilities and organizing weight management lectures). |  |  |  |  |  |
| D5 | I am aware of and can access information on government or organizational policies or services related to food safety and healthy diets (e.g., dietary guideline campaigns and the “three reductions” initiative: reducing salt, oil, and sugar). |  |  |  |  |  |
| D6 | I feel that my community, workplace, or school has performed well in implementing healthy diet-related measures (e.g., advocating healthy dietary concepts and disseminating knowledge about healthy eating). |  |  |  |  |  |
| D7 | I feel that my community, workplace, or school has taken concrete actions to promote healthy sleep schedules (e.g., organizing lectures on sleep science). |  |  |  |  |  |
| D8 | I believe that the government strictly enforces the smoking ban in public places. |  |  |  |  |  |
| D9 | I believe that the government has effectively implemented health education efforts on limiting alcohol consumption. |  |  |  |  |  |

*Note*: A, B, C, and D denote the four prespecified content domains: Individual Health and Behavior, Interpersonal Support, Built Environment Support, and Organizational Policy Support. The final 39-item MASE-HC comprises six empirically derived subscales: Mental Health, Physical Activity, Diet and Nutrition, Interpersonal Support, Built Environment Support, and Organizational Policy Support.

# Informed Consent Form

**Dear participant:**

Welcome to the study on the Multidimensional Assessment of Social-Ecological Healthy Communities. This study aims to collect information on your health-related behaviors and perceptions of the community environment and related social-ecological supports through a questionnaire, in order to provide evidence for improving community health ecology services. Please read the following information carefully before participating.

## I. Study Content

You will be asked to complete the Multidimensional Assessment Scale for Social-Ecological Healthy Communities. The questionnaire covers individual health and behavior, interpersonal support, built environment support, and organizational policy support.

## II. Participation

The questionnaire is expected to take approximately 10-15 minutes to complete and may be completed in paper or electronic form.

All personal information will be anonymized and used only for scientific research. No personally identifiable information will be disclosed.

## III. Your Rights

Participation is voluntary. You may refuse to participate or withdraw at any time without any adverse consequences. Your privacy will be protected: the data will be used only for academic research and will not be disclosed to third parties. By signing this consent form, you indicate that you understand the study and voluntarily agree to participate.

## IV. Participant Statement

I have read and understood the information above. I voluntarily agree to participate in this study and consent to the research team collecting and using my information in accordance with the study requirements.

This study is conducted by the School of Public Health, Anhui Medical University. The School of Public Health, Anhui Medical University will be the final institutional affiliation for presenting and publishing the research findings.

Participant signature: ____________________

Investigator signature: ____________________

Date of survey: Year 20____ Month ____ Day ____
